# Patient Preferences When Selecting Their Total Joint Arthroplasty Surgeon: A Multicenter Survey Study

**DOI:** 10.2106/JBJS.OA.25.00279

**Published:** 2025-12-10

**Authors:** Jake Laverdiere, Danielle Lonati, Swaroopa Vaidya, Gregory A. Panza, Antonia F. Chen, Mary Morcos, Yale A. Fillingham, Jessica H. Leipman, Molly A. Hartzler, Jenna Bernstein

**Affiliations:** 1 Connecticut Orthopaedic Institute, St. Vincent's Medical Center, Hartford Healthcare, Bridgeport, Connecticut; 2 Frank H. Netter MD School of Medicine, North Haven, Connecticut; 3 Department of Research, Hartford HealthCare, Hartford, Connecticut; 4 Department of Orthopaedics, University of Texas Southwestern, Dallas, Texas; 5 Harvard Combined Orthopaedics Residency Program, Boston, Massachusetts; 6 Department of Orthopaedic Surgery, Rothman Orthopaedic Institute, Sidney Kimmel Medical College of Thomas Jefferson University, Philadelphia, Pennsylvania; 7 Department of Orthopaedic Surgery and Rehabilitation, Wake Forest University, Winston-Salem, North Carolina; 8 Connecticut Orthopaedics, Trumbull, Connecticut

## Abstract

**Background:**

Surgeon selection can influence patient satisfaction, outcomes, and access in total joint arthroplasty (TJA). Awareness of patient preferences enables practices to enhance trust, optimize communication, and improve adherence. While referrals and word-of-mouth recommendations often outweigh online marketing, prospective multicenter research exploring how patients choose their TJA surgeon is limited. The aim of this study was to identify key decision-making factors and how they vary by demographics.

**Methods:**

This prospective, multicenter cross-sectional survey study included patients undergoing elective total joint arthroplasty at 4 high-volume institutions from January 2024 to 2025. Participants completed a survey on surgeon selection, including how they first heard of the surgeon, number of visits before deciding on surgery, and whether other surgeons were consulted. Patients rated 10 factors (1-10 scale) and selected the top 3 most important ones from a list of 7 factors.

**Results:**

Among 808 participants, most selected their surgeon through referrals (44.9%) or word-of-mouth recommendations (18.7%); only 8.4% cited online advertisements. Female patients rated surgeon gender (p = 0.010), ease of communication (p = 0.036), and time spent (P = 0.008) as more important than male patients. Minority patients prioritized gender (p = 0.003), race (p < 0.001), bedside manners (p = 0.013), time spent (p = 0.045), and implant type used (p = 0.004) more than White patients. Patients with a median household income between $30,000 and $70,000 rated insurance acceptance higher than those with household income higher than $300,000 (p = 0.007).

**Conclusions:**

Patients choose their arthroplasty surgeon primarily by way of referrals and word-of-mouth recommendations, underscoring the importance of trust. Female and minority patients value surgeon gender, race, and interpersonal skills, supporting culturally responsive care. Strengthening primary care physician engagement and community outreach may improve trust and promote equitable access.

## Introduction

Total joint arthroplasty (TJA) procedures are among the most frequently performed and impactful surgeries in orthopaedics. Annual procedure volume is projected to exceed 2 million by 2030, with estimated Medicare expenditure surpassing $50 billion^1^. As a result, patient satisfaction and surgeon selection have become crucial components of quality care and financial reimbursement^2^. The Centers for Medicare and Medicaid Services has tied reimbursement to patient experience, as captured by the Hospital Consumer Assessment of Healthcare Providers and Systems survey, which measures patients' perspectives on their care^3,4^.

To reach more patients, improve satisfaction, and grow their practices, many institutions use a variety of outreach strategies. Traditionally, surgeons have relied on word-of-mouth recommendations and referrals from primary care physicians. Newer advertising methods such as social media and digital marketing have gained considerable popularity. Although substantial financial investment and resources have been allocated toward these advertising techniques, prior research suggests that they may not adequately influence patient decision making^5,6^.

Despite existing literature on this topic, there is limited research eliciting how patients choose their TJA surgeon. One retrospective, single-center study found that patients were more likely to choose their TJA surgeon based on physician referral, board certification status, fellowship training, and insurance network^7^. The study provides valuable insight, however, is prone to bias and limited generalizability. Therefore, multicenter studies using a prospective approach to understanding patient decision making across a more diverse cohort are necessary. Furthermore, previous studies have not adequately explored how patient demographics or practice locations influence selection factors. Understanding patient preferences and decision-making processes may help to enhance patient experience and optimize surgical outcomes in orthopaedic practice. The aim of this prospective, multicenter study was to identify key factors patients consider when choosing their arthroplasty surgeon and whether these factors vary by patient characteristics.

## Materials and Methods

This prospective, multisite cross-sectional survey study included patients undergoing primary elective total hip arthroplasty (THA) or knee arthroplasty (TKA) at 4 high-volume orthopaedic institutions from January 1, 2024, through January 31, 2025. The study was approved by the institutional review board of the primary institution, with amendments for additional sites.

Patients aged 18 to 100 years scheduled for elective THA or TKA with a participating surgeon were invited to participate in the study. Following patient agreement, surveys were administered in paper or electronic format based on site preference. All completed surveys were entered into REDCap (REDCap), a centralized and secure data repository. The survey included demographic questions about the patient's age, race, ethnicity, gender, sexual orientation, insurance provider, primary language, highest level of education, occupation, household income, and zip code. Patient surveys also consisted of questions designed to address how patients chose their total joint arthroplasty surgeon. Finally, each survey listed 10 surgeon selection factors and asked patients to rank the top 3 most important ones.

### Data Analyses

Continuous data were checked for normality of distribution and that assumptions were met for the tests that were conducted. Categorical data are presented as frequency and percentages. Continuous variables are presented as means and standard deviation (SD) for parametric data or median and interquartile range (IQR) for nonparametric data. Categorical variables were compared using the χ^2^ analysis or Fisher exact test. Independent samples *t*-test and Mann-Whitney *U* test were used to analyze continuous variables for 2 groups and one-way Analysis of Variance or Kruskal-Wallis test for more than 2 groups. Bonferroni corrections for multiple comparisons were applied when appropriate. Analysis was performed using SPSS version 29.0, and statistical significance was defined as p < 0.05.

## Results

Associations between demographics factors and surgeon selection factors are provided in Table I. Among 808 participants (mean age: 66.4 years), most were female (62.4%), White/Caucasian (85.3%), English speaking (95.5%), retired, held a bachelor's degree, had median household income between $100,000 and $300,000, and on commercial insurance. Most patients first heard of their surgeon through referral, had seen them for less than 6 months, and decided on surgery at the first visit. Many had consulted at least 1 other orthopaedic surgeon.

**TABLE I:** Patient Demographics and Characteristics

Variable	Total Sample (N = 808)
Age (yr), mean ± SD	66.4 ± 10.5
Gender	
Female	504 (62.4)
Male	293 (36.3)
Did not respond	11 (1.4)
Race	
Asian	13 (1.6)
Black or African American	87 (10.8)
White or Caucasian	689 (85.3)
Other	20 (2.5)
Ethnicity	
Hispanic or Latino	27 (3.3)
Primary language spoken at home	
English	772 (95.5)
Spanish	6 (0.7)
Other	14 (1.7)
No response	16 (2.0)
Highest level of education	
Associate degree	68 (8.4)
Bachelor's degree	216 (26.7)
Doctorate degree	60 (7.4)
High school graduate or equivalent	95 (11.8)
Master’s degree	144 (17.8)
No schooling completed	2 (0.2)
Nursery school to eighth grade	3 (0.4)
Professional degree	34 (4.2)
Some college credit, no degree	114 (14.1)
Some high school, no diploma	16 (2.0)
Trade/technical/vocational training	50 (6.2)
No response	6 (0.7)
Current employment status	
Business owner	4 (0.5)
Employed	247 (30.6)
Retired	438 (54.2)
Self-employed	47 (5.8)
Unemployed	19 (2.4)
Other	42 (5.2)
No response	11 (1.4)
Level of activity at work	
Light or sedentary work	197 (24.4)
Mildly strenuous work	108 (13.4)
Moderately strenuous work	63 (7.8)
Heavy or manual labor	30 (3.7)
Not applicable	389 (48.1)
No response	21 (2.6)
Household income (US dollars)	
0-30k	94 (11.6)
>30-70k	155 (19.2)
>70-100k	147 (18.2)
>100-300k	273 (33.8)
>300k	57 (7.1)
No response	82 (10.1)
Primary insurance provider	
Commercial	385 (47.6)
Managed Medicare	84 (10.4)
Medicaid	14 (1.7)
Medicare	245 (30.3)
No response	80 (9.9)
How did you first hear about your surgeon?	136 (16.8)
I have been to this practice before Online	68 (8.4)
Other marketing	2 (0.2)
Referral from another doctor or provider	199 (24.6)
Referral from hospital/emergency room	15 (1.9)
Referral from primary care doctor or primary care provider	164 (20.3)
Word of mouth from friend or family	151 (18.7)
Other	63 (7.8)
No response	10 (1.2)
How long have you seen your surgeon?	
Less than 6 months	341 (42.2)
Six months to 1 year	165 (20.4)
More than 1 year	281 (34.8)
No response	21 (2.6)
How many visits prior to deciding to have joint replacement?	
Decision on first visit	393 (48.6)
Two visits	172 (21.3)
More than 2 visits	214 (26.5)
No response	29 (3.6)
Did you see other orthopaedic surgeons prior to this surgeon?	
Yes	419 (51.9)
No	376 (46.5)
No response	13 (1.6)
If applicable, how many other orthopaedic surgeons did you see?	n = 406
1	267 (65.8)
2	79 (19.5)
3	60 (14.8)

Associations between patient gender and surgeon selection factors are presented in Table II. Among 797 patients reporting gender (63.2% female), female patients more often learned of their surgeon through referral or word-of-mouth recommendations, while male patients were more often referred by a primary care physician (PCP) (p = 0.044). Female patients assigned greater importance to surgeon gender (p = 0.010) and ease of communication (p = 0.036) among their top 3 factors.

**TABLE II:** Patient Factors when Choosing Operative Arthroplasty Surgeon by Gender

Variable	Female (n = 504)	Male (n = 293)	Total (N = 797)	Statistic	p
General questions, frequency (%)				X^2^	
How did you first hear about your surgeon?	88 (17.5)	75 (25.6)*	163 (20.5)	14.45	0.044
Referral from primary care physician	127 (25.2)*	68 (23.2)	195 (24.5)		
Referral from another provider	9 (1.8)	6 (2.0)	15 (1.9)		
Referral from hospital/emergency room	78 (15.5)	56 (19.1)	134 (16.8)		
I have been to this practice before Word of mouth from friend or family	105 (20.8)*	44 (15.0)	149 (18.7)		
Online	47 (9.3)	21 (7.2)	68 (8.5)		
Other	43 (8.5)	22 (7.5)	65 (8.2)		
No response	7 (1.4)	1 (0.3)	8 (1.0)		
How many visits did you have prior to deciding to have joint replacement?				4.77	0.189
Decision on first visit	230 (45.6)	157 (53.6)	387 (48.6)		
Two visits	114 (22.6)	56 (19.1)	170 (21.3)		
More than 2 visits	142 (28.2)	70 (23.9)	212 (26.6)		
No response	18 (3.6)	10 (3.4)	28 (3.5)		
Did you see other orthopaedic surgeons prior to this surgery?				0.36	0.836
Yes	264 (52.4)	148 (50.5)	412 (51.7)		
No	232 (46.0)	141 (48.1)	373 (46.8)		
No response	8 (1.6)	4 (1.4)	12 (1.5)		
Rating of importance when choosing surgeon (0-10 scale), median, IQR				U	
I feel I can trust the surgeon	10.0, 0.0	10.0, 0.0	10.0, 0.0	67,841.0	0.719
The surgeon's gender	0.0, 1.0	0.0, 0.0	0.0, 0.0	60,062.0	0.010
The surgeon's race	0.0, 0.0	0.0, 0.0	0.0, 0.0	63,223.0	0.337
I know someone who had a good result with the surgeon	4.0, 10.0	5.0, 10.0	5.0, 10.0	53,741.0	0.572
My doctor recommended the surgeon	8.0, 10.0	8.0, 10.0	8.0, 10.0	60,259.5	0.783
I do not have time to find another surgeon	0.0, 0.0	0.0, 0.0	0.0, 0.0	55,219.0	0.664
The surgeon accepts my insurance	10.0, 5.0	10.0, 5.0	10.0, 5.0	63,607.5	0.826
The hospital or practice has a good reputation	10.0, 1.0	10.0, 1.0	10.0, 1.0	63,602.0	0.273
The technology this surgeon uses in surgery	10.0, 5.0	10.0, 3.0	10.0, 4.0	59,856.0	0.334
The implants this surgeon uses in surgery	9.0, 8.0	9.0, 5.0	9.0, 6.0	57,590.5	0.173
The 3 most important factors when choosing your surgeon, frequency (%)				X^2^	
Bedside manner	220 (43.7)	111 (37.9)	331 (41.5)	2.5	0.111
Time spent with the patient	266 (52.8)	126 (43.0)	392 (49.2)	7.1	0.008
Technical skills	351 (69.6)	215 (73.4)	566 (71.0)	1.3	0.262
Ease of communication with the surgeon’s office	224 (44.4)	108 (36.9)	332 (41.7)	4.4	0.036
The implants the surgeon uses in surgery	86 (17.1)	66 (22.5)	152 (19.1)	3.6	0.058
The technology the surgeon uses in surgery	189 (37.5)	122 (41.6)	311 (39.0)	1.3	0.248
Location (e.g., ASC vs inpatient setting, reputation of hospital)	163 (32.3)	87 (29.7)	250 (31.4)	0.6	0.437

*Statistically significant (p = 0.044). ASC = ambulatory surgical center.

Note: Gender was not declared by 11 patients.

Associations between patient race and surgeon selection factors are provided in Table III. Among the 808 patients (14.7% minority), minority patients were more likely to select “other” or provide no response, while White patients more frequently cited online sources when asked how they learned of their surgeon (p = 0.002). Minority patients rated surgeon gender (P = 0.003), race (p < 0.001), technology (p = 0.008), and implant type (p < 0.001) higher, while White patients rated insurance type higher (p < 0.001). For top 3 factors, minority patients more often selected bedside manners (p = 0.013), time spent (p = 0.045), and implant type (p = 0.004), whereas White patients prioritized technical skills (p < 0.001).

**TABLE III:** Patient Factors when Choosing Operative Arthroplasty Surgeon by Race: Caucasian or White vs. Minority

Variable	Minority (n = 119)	White/Caucasian (n = 689)	Total (N = 808)	Statistic	p
General questions, frequency (%)				X^2^	
How did you first hear about your surgeon?	31 (26.1)	133 (19.3)	164 (20.3)	22.65	0.002
Referral from PCP	23 (19.3)	176 (25.5)	199 (24.6)		
Referral from another provider	2 (1.7)	13 (1.9)	15 (1.9)		
Referral from hospital/emergency room	21 (17.6)	115 (16.7)	136 (16.8)		
I have been to this practice before Word of mouth from friend or family	18 (15.1)	133 (19.3)	151 (18.7)		
Online	4 (3.4)	64 (9.3)*	68 (8.4)		
Other	15 (12.6)*	50 (7.3)	65 (8.0)		
No response	5 (4.2)*	5 (0.7)	10 (1.2)		
How many visits did you have prior to deciding to have joint replacement?				7.03	0.071
Decision on first visit	54 (45.4)	339 (49.2)	393 (48.6)		
Two visits	18 (15.1)	154 (22.4)	172 (21.3)		
More than 2 visits	42 (35.3)	172 (25.0)	214 (26.5)		
No response	5 (4.2)	24 (3.5)	29 (3.6)		
Did you see other orthopaedic surgeons prior to this surgery?				0.08	0.963
Yes	63 (52.9)	356 (51.7)	419 (51.9)		
No	54 (45.4)	322 (46.7)	376 (46.5)		
No response	2 (1.7)	11 (1.6)	13 (1.6)		
Rating of importance when choosing surgeon (0-10 scale), median, IQR				U	
I feel I can trust the surgeon	10.0, 0.0	10.0, 0.0	10.0, 0.0	36,468.0	0.310
The surgeon's gender	0.0, 5.0	0.0, 0.0	0.0, 1.0	31,206.5	0.003
The surgeon's race	0.0, 2.0	0.0, 0.0	0.0, 0.0	29,878.0	<0.001
I know someone who had a good result with the surgeon	5.0, 10.0	4.5, 10.0	5.0, 10.0	29,820.0	0.438
My doctor recommended the surgeon	6.5, 10.0	8.0, 10.0	8.0, 10.0	33,696.5	0.659
I do not have time to find another surgeon	0.0, 0.0	0.0, 0.0	0.0, 0.0	28,804.0	0.210
The surgeon accepts my insurance	10.0, 0.0	10.0, 6.0	10.0, 5.0	27,351.0	<0.001
The hospital or practice has a good reputation	10.0, 0.0	10.0, 1.0	10.0, 1.0	33,414.0	0.111
The technology this surgeon uses in surgery	10.0, 2.0	9.0, 4.0	10.0, 4.0	29,796.0	0.008
The implants this surgeon uses in surgery	10.0, 5.0	9.0, 6.0	9.0, 6.0	27,643.0	<0.001
The 3 most important factors when choosing your surgeon, frequency (%)				X^2^	
Bedside manner	62 (52.1)	275 (39.9)	337 (41.7)	6.2	0.013
Time spent with the patient	69 (58.0)	331 (48.0)	400 (49.5)	4.0	0.045
Technical skills	67 (56.3)	505 (73.3)	572 (70.8)	14.2	<0.001
Ease of communication with the surgeon’s office	54 (45.4)	281 (40.8)	335 (41.5)	0.8	0.348
The implants the surgeon uses in surgery	34 (28.6)	119 (17.3)	153 (18.9)	8.4	0.004
The technology the surgeon uses in surgery	38 (31.9)	277 (40.2)	315 (39.0)	2.9	0.088
Location (e.g., ASC vs inpatient setting, reputation of hospital)	35 (29.4)	217 (31.5)	252 (31.2)	0.2	0.651

*Statistically significant (p = 0.044). ASC = ambulatory surgical center, PCP = primary care physician.

Note: Minority race includes Asian, Black or African American, or Other.

Associations between geographic location and surgeon selection factors are summarized in Table IV. Among the 808 participants, 25.4% were from Pennsylvania (PA), 24.8% from North Carolina (NC), 4.4% from Connecticut (CT), and 45.4% from Massachusetts (MA). Geographic differences existed in how patients first heard of their surgeon (p < 0.001), decided on surgery at the first visit (p = 0.028), and prior consultations (p < 0.001). Patients in PA, NC, and CT valued insurance acceptance and implant type more than MA (p < 0.001), while patients in MA valued surgeon gender (p = 0.002) and hospital reputation (p < 0.001). Patients in CT and MA more often cited insufficient time to find another surgeon compared with PA and NC (p < 0.001). In NC and CT, knowing someone with a favorable outcome was more valuable than that in MA (p < 0.001), and in CT more than PA (p < 0.001). Technology was valued significantly more in PA and CT than that in MA (p < 0.001), and more in NC than in CT (p < 0.001). Patients in PA valued physician recommendations more than that in MA (p = 0.003). In NC, bedside manners were more often ranked among the top 3 factors compared with MA (p < 0.001), while patients in MA prioritized time spent (p = 0.004) and facility location (p = 0.009).

**TABLE IV:** Patient Factors when Choosing Operative Arthroplasty Surgeon by Site Location

Variable	Philadelphia (n = 205)	North Carolina (n = 200)	Connecticut (n = 36)	Massachusetts (n = 367)	Total (N = 808)	X^2^	p
General questions, frequency (%)							
How did you first hear about your surgeon?	44 (21.5)^a^	42 (21.0)^b^	9 (25.0)^a,b^	69 (18.8)^a,b^	164 (20.3)	94.56	<0.001
Referral from PCP	50 (24.4)	51 (25.5)	4 (11.1)	94 (25.6)	199 (24.6)		
Referral from another provider	0 (0.0)^a^	8 (4.0)^b^	0 (0.0)^a,b^	7 (1.9)^a,b^	15 (1.9)		
Referral from hospital/emergency room	60 (29.3)^a^	29 (14.5)^b,c^	10 (27.8)^a,c^	37 (10.1)^b^	136 (16.8)		
I have been to this practice before Word of mouth from friend or family	25 (12.2)^a^	52 (26.0)^b^	7 (19.4)^a,b^	67 (18.3)^a,b^	151 (18.7)		
Online	4 (2.0)^a^	8 (4.0)^a^	2 (5.6)^a,b^	54 (14.7)^b^	68 (8.4)		
Other	20 (9.8)	9 (4.5)	3 (8.3)	33 (9.0)	65 (8.0)		
No response	2 (1.0)	1 (0.5)	1 (2.8)	6 (1.6)	10 (1.2)		
How many visits did you have prior to deciding to have joint replacement?						14.13	0.028
Decision on first visit	116 (56.6)^a^	122 (61.0)^a^	9 (25.0)^b^	146 (39.8)^b^	393 (48.6)		
Two visits	39 (19.0)	36 (18.0)	12 (33.3)	85 (23.2)	172 (21.3)		
More than 2 visits	49 (23.9)	41 (20.5)	14 (38.9)	110 (30.0)	214 (26.5)		
No response	1 (0.5)	1 (0.5)	1 (2.8)	26 (7.1)	29 (3.6)		
Did you see other orthopaedic surgeons prior to this surgery?						53.94	<0.001
Yes	102 (49.8)^a,b^	90 (45.0)^b^	19 (52.8)^a,b^	208 (56.7)^a^	419 (51.9)		
No	103 (50.2)	107 (53.5)	16 (44.4)	150 (40.9)	376 (46.5)		
No response	0 (0.0)	3 (1.5)	1 (2.8)	9 (2.5)	13 (1.6)		
Rating of importance when choosing surgeon (0-10 scale), median, IQR							
I feel I can trust the surgeon	10.0, 0.0^a^	10.0, 0.0^b^	10.0, 0.0^a,b^	10.0, 0.0^a^	10.0, 0.0	22.4	<0.001
The surgeon’s gender	0.0, 0.0^a,b^	0.0, 0.0^b^	0.0, 5.0^a,b^	0.0, 2.0^c^	0.0, 1.0	15.3	0.002
The surgeon’s race	0.0, 0.0	0.0, 0.0	0.0, 1.0	0.0, 0.0	0.0, 0.0	6.3	0.099
I know someone who had a good result with the surgeon	5.0, 10.0^a,b,c^	7.0, 10.0^c,d^	10.0, 8.0^d^	3.0, 9.0^a^	5.0, 10.0	17.9	<0.001
My doctor recommended the surgeon	9.0, 10.0^a^	8.0, 10.0^a,b^	7.0, 10.0^a,b^	7.0, 10.0^b^	8.0, 10.0	13.7	0.003
I do not have time to find another surgeon	0.0, 0.0^a^	0.0, 0.0^a^	0.0, 4.0^b^	0.0, 1.0^c^	0.0, 0.0	33.2	<0.001
The surgeon accepts my insurance	10.0, 4.0^a^	10.0, 3.0^b^	10.0, 5.0^b^	9.0, 9.0^b^	10.0, 5.0	37.9	<0.001
The hospital or practice has a good reputation	10.0, 0.0^a^	10.0, 0.0^b^	10.0, 0.0^b^	10.0, 2.0^b^	10.0, 1.0	52.3	<0.001
The technology this surgeon uses in surgery	10.0, 2.0^a,b^	10.0, 5.0^b,c^	10.0, 1.0^a^	9.0, 5.0^c^	10.0, 4.0	30.1	<0.001
The implants this surgeon uses in surgery	10.0, 3.0^a^	10.0, 9.0^b^	10.0, 4.0^b^	8.0, 7.0^b^	9.0, 6.0	36.3	<0.001
The 3 most important factors when choosing your surgeon, frequency (%)							
Bedside manner	87 (42.4)^a,b^	105 (52.5)^b^	18 (50.0)^a,b^	127 (34.6)^a^	337 (41.7)	18.3	<0.001
Time spent with the patient	95 (46.3)^a,b^	82 (41.0)^b^	23 (63.9)^a,b^	200 (54.5)^a^	400 (49.5)	13.2	0.004
Technical skills	144 (70.2)	129 (64.5)	26 (72.2)	272 (74.4)	572 (70.8)	6.2	0.103
Ease of communication with the surgeon’s office	83 (40.5)	96 (48.0)	12 (33.3)	144 (39.2)	335 (41.5)	5.3	0.149
The implants the surgeon uses in surgery	50 (24.4)	33 (16.5)	9 (25.0)	61 (16.6)	153 (18.9)	6.9	0.076
The technology the surgeon uses in surgery	85 (41.5)	70 (35.0)	17 (47.2)	143 (39.0)	315 (39.0)	2.9	0.409
Location of surgery (e.g., ASC vs inpatient setting, hospital reputation)	67 (32.7)^a,b^	47 (23.5)^b^	7 (19.4)^a,b^	131 (35.7)^a^	252 (31.2)	11.5	0.009

ASC = ambulatory surgical center, PCP = primary care physician.

Note: Differing subscript letters denote where significant difference occurred between groups at the 0.05 level following Bonferroni adjustment.

Associations between median household income and surgeon selection factors are detailed in Table V. Among 808 participants, median household income groups were $0 to $30,000 (11.6%), $30,000 to $70,000 (19.2%), $70,000 to $100,000 (18.2%), $100,000 to $300,000 (33.8%), and >$300,000 (7.1%); 10.1% did not respond. Patients in the $0 to $30,000 group prioritized surgeon gender and race (both p < 0.001) and, more frequently, cited lack of time to find another surgeon (p = 0.014) as a contributing factor. The $30,000 to $70,000 group emphasized insurance acceptance (p = 0.007) and implant type (p = 0.001) compared with higher income groups. The $70,000 to $100,000 group valued trust in the surgeon more than the $30,000 to $70,000 group (p = 0.029). The $100,000 to $300,000 group valued hospital reputation more than the $30,000 to $70,000 group (p = 0.011) and were more likely to list technical skills and ease of communication among their top 3 factors (p < 0 0.001).

**TABLE V:** Patient Factors when Choosing Operative Arthroplasty Surgeon by Median Household Income

Variable	$0 -$30,000 (n = 94)	>$30,000 - $70,000 (n = 155)	>$70,000 -$100,000 (n = 147)	>$100,000 -$300,000 (n = 273)	>$300,000 (n = 57)	X^2^	p
General questions, frequency (%)							
How did you first hear about your surgeon?	24 (25.5)	29 (18.7)	58 (21.2)	7 (12.3)	0 (0.0)	34.7	0.179
Referral from PCP	31 (33.0)	38 (24.5)	35 (23.8)	56 (20.5)	16 (28.1)		
Referral from another provider	3 (3.2)	5 (3.2)	1 (0.7)	3 (1.1)	1 (1.8)		
Referral from hospital/emergency room	10 (10.6)	27 (17.4)	29 (19.7)	44 (16.1)	8 (14.0)		
I have been to this practice before Word of mouth from friend or family	11 (11.7)	31 (20.0)	31 (21.1)	52 (19.0)	15 (26.3)		
Online	7 (7.4)	10 (6.5)	10 (6.8)	30 (11.0)	7 (12.3)		
Other	5 (5.3)	15 (9.7)	11 (7.5)	27 (9.9)	3 (5.3)		
No response	3 (3.2)	0 (0.0)	1 (0.7)	3 (1.1)	0 (0.0)		
How many visits did you have prior to deciding to have joint replacement?						13.8	0.312
Decision on first visit	41 (43.6)	87 (56.1)	78 (53.1)	129 (47.3)	23 (40.4)		
Two visits	18 (19.1)	32 (20.6)	24 (16.3)	64 (23.4)	14 (24.6)		
More than 2 visits	33 (35.1)	34 (21.9)	40 (27.2)	69 (25.3)	18 (31.6)		
No response	2 (2.1)	2 (1.3)	5 (3.4)	11 (4.0)	2 (3.5)		
Did you see other orthopaedic surgeons prior to this surgery?						7.2	0.514
Yes	48 (51.1)	82 (52.9)	69 (46.9)	141 (51.6)	36 (63.2)		
No	44 (46.8)	72 (46.5)	77 (52.4)	127 (46.5)	21 (36.8)		
No response	2 (2.1)	1 (0.6)	1 (0.7)	5 (1.8)	0 (0.0)		
Rating of importance when choosing surgeon (0-10 scale), median, IQR							
I feel I can trust the surgeon	10.0, 0.0^a,b^	10.0, 0.0^a^	10.0, 1.0^b^	10.0, 0.0^a,b^	10.0, 0.0^a,b^	10.8	0.029
The surgeon’s gender	0.0, 9.0^a^	0.0, 2.0^b^	0.0, 0.0^b^	0.0, 1.0^b^	0.0, 1.0^a,b^	19.4	<0.001
The surgeon’s race	0.0, 5.0^a^	0.0, 0.0^a,b^	0.0, 0.0^b^	0.0, 0.0^b^	0.0, 0.0^b^	19.9	<0.001
I know someone who had a good result with the surgeon	0.0, 10.0	5.0, 10.0	3.0, 10.0	5.5, 10.0	7.0, 10.0	6.5	0.165
My doctor recommended the surgeon	9.0, 10.0	8.0, 10.0	9.0, 10.0	8.0, 10.0	8.0, 10.0	3.2	0.523
I do not have time to find another surgeon	0.0, 2.0^a^	0.0, 0.0^a,b^	0.0, 0.0^b^	0.0, 1.0^a,b^	0.0, 0.0^a,b^	12.6	0.014
The surgeon accepts my insurance	10.0, 3.0^a,b^	10.0, 4.0^a^	10.0, 4.0^a,b^	10.0, 6.0^a,b^	7.0, 7.0^b^	14.2	0.007
The hospital or practice has a good reputation	10.0, 0.0^a,b^	10.0, 0.0^b^	10.0, 1.0^a,b^	10.0, 1.0^a^	10.0, 1.0^a,b^	13.1	0.011
The technology this surgeon uses in surgery	10.0, 3.0	10.0, 3.0	10.0, 3.0	9.0, 4.0	10.0, 5.0	9.1	0.059
The implants this surgeon uses in surgery	10.0, 5.0^a,b^	10.0, 5.0^a^	9.0, 6.0^a,b^	8.0, 6.0^b^	7.0, 10.0^b^	17.7	0.001
The 3 most important factors when choosing your surgeon, frequency (%)							
Bedside manner	43 (45.7)	73 (47.1)	56 (38.1)	109 (39.9)	21 (36.8)	4.2	0.382
Time spent with the patient	55 (58.5)	69 (44.5)	78 (53.1)	126 (46.2)	31 (54.4)	7.1	0.130
Technical skills	56 (59.6)^a^	99 (63.9)^a^	106 (72.1)^a,b^	215 (78.8)^b^	44 (77.2)^a,b^	19.0	<0.001
Ease of communication with the surgeon’s office	45 (47.9)^a,b,c^	75 (48.4)^b,c^	76 (51.7)^c^	92 (33.7)^a^	16 (28.1)^a,b^	21.9	<0.001
The implants the surgeon uses in surgery*	18 (19.1)	35 (22.6)	16 (10.9)	59 (21.6)	7 (12.3)	10.6	0.032
The technology the surgeon uses in surgery	34 (36.2)	60 (38.7)	51 (34.7)	115 (42.1)	26 (45.6)	3.6	0.466
Location of surgery (e.g., ASC vs inpatient setting, hospital reputation)	27 (28.7)	48 (31.0)	46 (31.3)	89 (32.6)	17 (29.8)	0.6	0.966

Note: Eighty-two participants did not report their median household income.

Differing subscript letters denote where significant difference occurred between groups at the 0.05 level following Bonferroni adjustment.

* No significant difference between groups following Bonferroni adjustment. PCP = primary care physician.

## Discussion

This study sought to investigate factors patients consider to be the most important when selecting their arthroplasty surgeon. Trust emerged as the top factor, with a median rating of 10 out of 10 across all groups. Most patients selected their surgeon through referrals from healthcare providers or word-of-mouth recommendations from family or friends. Certain selection factors were more important for patients based on their gender and race. For example, a surgeon’s gender was more important for female and minority patients. Minority patients also prioritized surgeon race, time spent with patients, use of technology, and implant choices. Finally, patients with a lower median household income (>$30,000 to $70,000) valued surgeons who accepted their insurance more than patients with the highest median household income (>$300,000).

The emphasis on referrals and word-of-mouth recommendations aligns with prior literature^5-8^. This likely reflects the role of trust in the patient-physician relationship, as physicians are perceived as authority figures due to their extensive training and expertise. Physician referrals represent meaningful endorsement, conveying trust and professional respect, both of which are highly valued by patients^9^. While referrals are not always required to see a specialist, such as an orthopaedic surgeon, many insurance providers mandate them. Patients with Medicaid are significantly more likely to be denied appointments without a referral than those with private insurance^10^. Furthermore, patients with joint pain often present first to general practitioners and are subsequently referred to an orthopaedic surgeon^11^. Hospital systems may therefore benefit from strengthening PCP relationships and emphasizing referral-based outreach over direct-to-consumer advertisement strategies.

Female and minority patients may experience vulnerability in healthcare settings and subsequently prioritize surgeon gender to enhance comfort. Similarly, race was an important factor for minority patients, suggesting that racial concordance can foster trust and reassurance. Gender and racial concordance has been shown to improve communication, reduce bias, strengthen rapport, and improve outcomes^12-14^. Patients who share race or gender identity with their surgeon often report greater trust and comfort, with female patients tending to rate female surgeons more favorably^15,16^. Patient satisfaction also increases when their physician’s racial or ethnic background aligns with their own, emphasizing the role of racial concordance in optimizing patient experience^17^. Institutional efforts to enhance diversity and cultural competency can help foster positive relationships and comfort for all populations.

This study is also the first to report time spent with patients, use of advanced technology, and implant selection as important factors for minority patients. Historical and systemic inequities may fuel distrust among minority patients, making personalized attention especially valuable. Spending more time can signify respect and conveys a genuine interest in a patient’s concerns^18^. Longer, more thoughtful interactions may help counteract previous negative healthcare experiences and build trust. Substantial research has demonstrated that minority groups often receive lower-quality care, even when insurance status is comparable^19,20^. Minority and lower-income patients may therefore prioritize qualities reflecting respect, cultural understanding, and high-quality care. Institutions that prioritize surgeon diversity, cultural competency, and clear communication could help reduce disparities and improve patient experience. Demographic-specific outreach may be essential to achieving equitable, patient-centered care in total joint arthroplasty.

Consistent with prior research^5,6,21^, this study found that online marketing and social media were less influential for patient decision making. Despite the growing use among surgeons, social media may be limited in its influence due to the older average age of TJA patients (65-67 years)^22,23^. For instance, 1 study investigating the importance of word-of-mouth versus online engagement when selecting a sports medicine surgeon found that increased age, greater than 65 years, was associated with a decreased knowledge and general use of social media platforms^24^. Misinformation on social media may also undermine trust^25^. Studies assessing the influence of orthopaedic surgeons on social media suggest patients respond positively to educational content and testimonials rather than to self-promotional content^23^. Therefore, institutions should re-evaluate cost-effectiveness of conventional marketing and invest in strategies that build trust and interpersonal connections, such as patient ambassador programs, testimonials, physician meet-and-greets, and PCP outreach.

Our findings demonstrate how geographical location has a meaningful influence on patient preference. Patients in Pennsylvania and Connecticut valued insurance acceptance and technology use, while those in Massachusetts prioritized hospital reputation and time spent with the patient. Furthermore, patients in North Carolina prioritized good bedside manners.

While the study was limited to select regions, findings suggest that geographic location may influence patient preferences, potentially due to differences in healthcare systems, insurance, and referral practices. Further studies with broader inclusion of institutions serving diverse populations is needed to improve generalizability and assess whether these trends persist nationwide. Understanding these patterns can help surgeons adapt to effectively meet the expectations of their community.

### Limitations

There are several limitations inherent to the study and its design that should be considered. Although the study design minimized recall bias, patients may have unconsciously aligned their responses with their current surgeon. The sample lacked diversity, with most participants being English-speaking, White, commercially insured, and highly educated, limiting generalizability to underrepresented populations. While the aim of this study was to address gaps in the literature, the findings primarily reflect demographics of those who chose to participate.

Despite efforts to recruit surgeons from diverse practice settings across the United States, participation was limited to institutions that agreed to take part. The large data set represents most patients within those practices; however, due to the nature of the study, we were unable to assess demographic differences between participants and nonparticipants. Future studies would benefit from tracking response rates and assessing demographic differences.

## Conclusion

This study highlights the complex and multifactorial considerations patients face when selecting an arthroplasty surgeon. Trust emerged as the most influential factor, with most patients relying on referrals and word-of-mouth recommendations. Female and minority patients prioritized surgeon gender, race, time spent, and technology use, underscoring the importance of trust, communication, and cultural comfort in surgical decision making. These findings support PCP engagement and community outreach as effective strategies for building trust and promoting equitable access to care. Future research should incorporate more diverse populations and explore whether aligning care with patient preferences improves satisfaction, reduces disparities, and enhances outcomes.
